# A case report of aspergillosis accompanied by saccular bronchodilation after bronchial thermoplasty in a 19-year-old woman

**DOI:** 10.1186/s12890-020-01352-y

**Published:** 2020-11-26

**Authors:** Shinji Sasada, Kenshiro Ohmura, Tomoyo Oguri, Yutaro Fujimoto, Saori Murata, Yumi Tsuchiya, Kota Ishioka, Saeko Takahashi, Morio Nakamura, Masahiro Kaji

**Affiliations:** 1 Department of Respiratory Medicine, Tokyo Saiseikai Central Hospital, 1-4-17 Mita, Minato-ku, Tokyo, 108-0073 Japan; 2 Department of Thoracic Surgery, Tokyo Saiseikai Central Hospital, Tokyo, Japan; 3 Department of Clinical Oncology, St. Marianna University School of Medicine, Kawasaki, Japan

**Keywords:** Aspergillosis, Saccular bronchodilation, Bronchial thermoplasty, Severe asthma, Case report

## Abstract

**Background:**

Fungal infections are rarely reported as a complication of bronchial thermoplasty (BT) in patients without immunosuppressive comorbidity.

**Case presentation:**

A 19-year-old woman college student was admitted to our hospital owing to uncontrolled severe asthma despite using the maximum dose of steroid inhalation. She experienced asthmatic attacks more frequently while cheerleading, which is an extracurricular activity. She received BT because she wanted to continue cheerleading. After the second BT session, she developed more sputum and cough. During the third session, white secretion and saccular bronchodilation appeared in the left lower bronchus. Aspergillus fumigatus was detected in the culture of the bronchial lavage sample, and saccular bronchodilation in the affected bronchus was observed on computed tomography (CT). Five months after the start of oral itraconazole, her subjective symptoms as well as her CT findings improved. Her asthma condition improved enough for the patient to continue cheerleading without exacerbation.

**Conclusions:**

It is necessary to consider the possibility of respiratory tract infections including fungal infections after BT. Detailed observations of the entire bronchus and sample collection for microbial culture are highly recommended.

## Background

Bronchial thermoplasty (BT) is a medical procedure in which bronchoscopy is used and controlled thermal energy is applied to the bronchial wall to decrease the smooth muscles. This procedure is also effective for the treatment of severe asthma. Bronchial edema and radiological changes are commonly known major complications of BT, but infections are rarely reported. The AIR2 trial reported that only one patient with lower respiratory tract infection required hospitalization in the BT group [1]. Here, we present a case of aspergillosis after BT in a young asthmatic patient.

## Case presentation

The patient was a 19-year-old woman with no comorbidities other than asthma. She was a non-smoker. She had been treated for severe asthma with high doses of inhaled corticosteroids plus long-acting β2-agonists, antileukotriene, theophylline, and antihistamine. The patient participated in cheerleading as an extracurricular activity at her college. During cheerleading, she experienced asthmatic attacks, which became increasingly frequent, and she therefore sought medical consultation. The patient wanted to continue cheerleading and enquired about strengthening her treatment. However, she did not want to take regular corticosteroids or antibody agents. She refused biological treatment owing to the cost associated with long-term continuation and the desire to have children. Therefore, the patient decided to undergo BT.

**Fig. 1:**
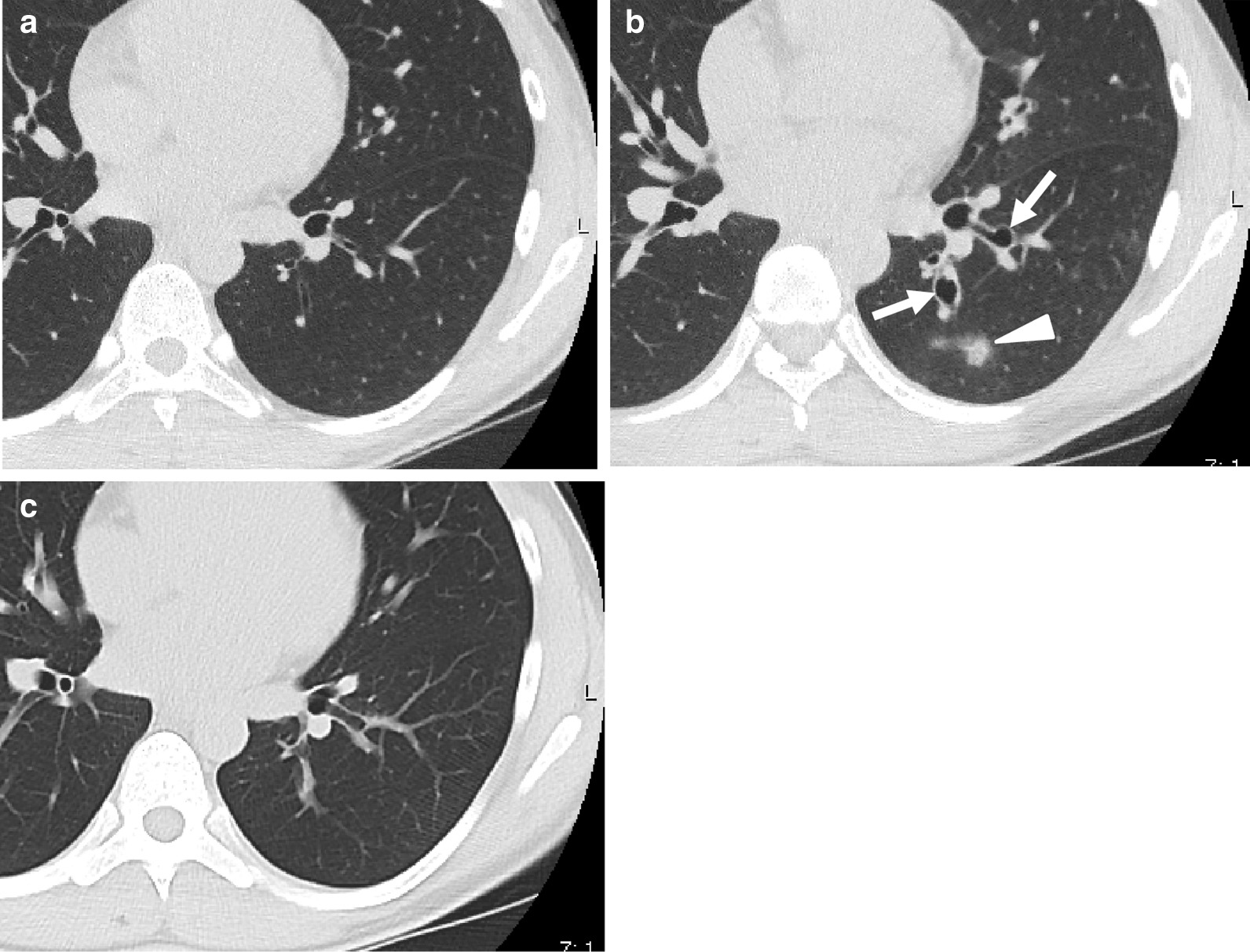
Chest computed tomography images. **a** Left lower lobe with thickened bronchial wall before bronchial thermoplasty (BT). **b** Saccular bronchodilation of left lower bronchus (B9, B10) (arrows) and nodular consolidation (triangle) are seen 3 weeks after BT. **c** Bronchodilation and consolidation are improved 5 months after the start of oral itraconazole administration

**Fig. 2:**
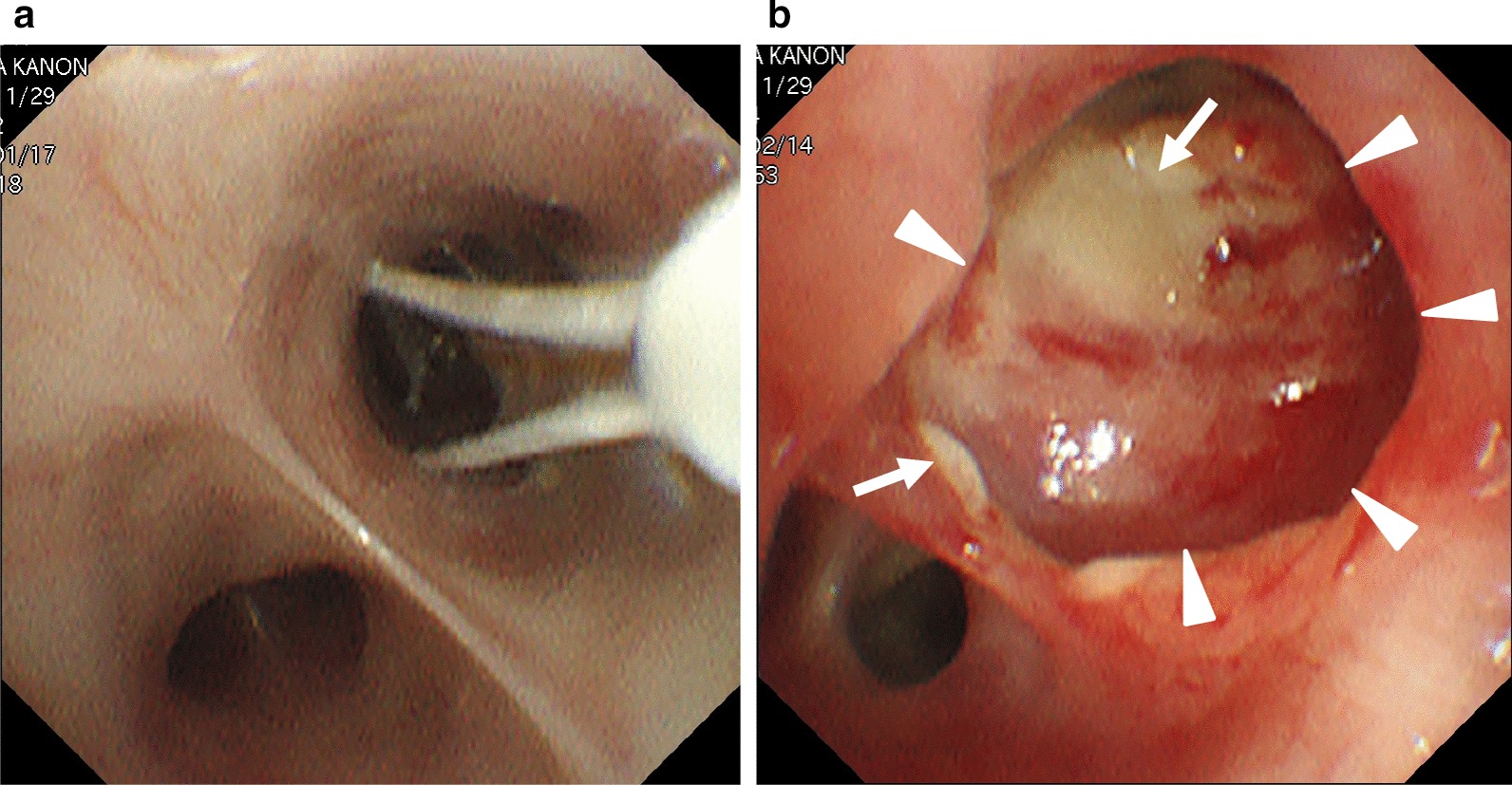
Bronchoscopic images. **a** Bronchial thermoplasty (BT) activation to left B9. **b** White sputum (arrows) and saccular bronchodilation (triangles) are seen 3 weeks after BT (B)

Chest computed tomography (CT) showed a thickened bronchial wall (Fig. 1a); the percentage forced expiratory volume (%FEV) was 86.2%, and exhaled nitric oxide was 45 ppm. Blood test results revealed the following: 515 U/mL, IgE; 8900/μL, white blood cell count; and 143/μL, eosinophils. The patient received 30 mg/day prednisolone for 3 days before BT treatment and continued it for 1 day post-BT treatment. The sessions for the right and left lower lobe were properly completed at 3-week intervals, and the BT activation numbers were 31 and 24 times, respectively (Fig. 2a).

After the second BT session, she developed more sputum and cough. During the third session, white secretion and saccular bronchodilation appeared in the left lower bronchus (B9 and B10) (Fig. 2b). At the same time, bronchial washing was performed. Her CT also showed saccular bronchodilation in the affected bronchus (Fig. 1b). Aspergillus fumigatus was detected in the bronchial lavage culture. Oral itraconazole administration was started, and serum β-d-glucan and aspergillus antigen were found to be negative. Five months later, the subjective symptoms and CT findings improved (Fig. 1c). Although respiratory function remained unchanged (%FEV, 88%), exhaled nitric oxide reduced to 15 ppm. After BT, the inhaled steroid dose was reduced owing to a remarkable improvement in asthma symptoms. The patient was able to continue her cheerleading activity without exacerbations.

## Discussion and conclusions

Previous studies have evaluated the efficacy and safety of BT in patients with severe asthma. The AIR2 study showed that BT improved asthma-related quality of life (QOL) and significantly controlled the frequency of severe exacerbations [1]. In addition, the efficacy of BT can last for at least 5 years [2]. Moreover, Japanese patients with asthma had improved QOL, less exacerbations, less symptoms, and less obstructive pulmonary function after BT [3]. However, some complications associated with BT have been reported. Burn et al. reported that their BT patients experienced adverse events more frequently than those described in previous clinical trials [4]. Iikura et al. reported a case of aspergillus and nocardia infection after BT [5]: the patient was a 35-year-old man who regularly received systemic corticosteroids, which resulted in chronic immunodeficiency. In our case, the patient did not receive regular systemic corticosteroids, and the prophylactic steroid dose was 30 mg/body weight, which was less than the recommended dose of 50 mg/body weight in Japan [6]. In our experience, high-dose inhaled corticosteroids and minimal prophylactic systemic corticosteroids may cause fungal infections even in the absence of immunosuppressive comorbidities.

In addition, heat energy from BT may cause tissue fragility that predisposes the patient to respiratory tract infections [7]. However, in this case, the activation number for the left lower lobe was 24 times, which was much less than the average [6], and it developed while the damage to the respiratory tract was kept to a minimum. Potential respiratory aspergillosis has been previously reported in asthmatic patients who received inhaled corticosteroids [8, 9]. Hypothetically, it is considered that the mucosal defense is reduced following BT; thus, the pre-existing aspergillus molds become engrafted. In this case, aspergillosis was not tested for before performing BT. From this experience, pre-screening of chronic infections of the respiratory tract should be carried out. On the other hand, serum β-D-glucan and the aspergillus antigen were negative after occurring aspergillosis, the reason for the negative may be that the infection remained localized and the medication was started early. If the aspergillosis was not diagnosed and treated early, the bronchial deformity might have become irreversible.

We report a case of aspergillosis infection accompanied by saccular bronchodilation after BT. Regardless of the strength of asthma treatment and the absence of an immunosuppressive comorbidity, it is necessary to consider the possibility of respiratory tract infections, including fungal infections. Detailed observations of the entire bronchus and sample collection for microbial culture are highly recommended.

## Data Availability

All data and material are available for sharing if needed.

## Abbreviations

BT: Bronchial thermoplasty
CT: Chest computed tomography
QOL: Quality of life
FEV: Forced expiratory volume
